# The Flo Adhesin Family

**DOI:** 10.3390/pathogens10111397

**Published:** 2021-10-28

**Authors:** Ronnie G. Willaert, Yeseren Kayacan, Bart Devreese

**Affiliations:** 1 Research Group Structural Biology Brussels (SBB), Vrije Universiteit Brussel (VUB), 1050 Brussels, Belgium; yeserenk@gmail.com; 2 Alliance Research Group VUB-UGent NanoMicrobiology (NAMI), 1050 Brussels, Belgium; Bart.Devreese@ugent.be; 3 International Joint Research Group VUB-EPFL NanoBiotechnology & NanoMedicine (NANO), Vrije Universiteit Brussel (VUB), 1050 Brussels, Belgium; 4 Ecole Polytechnique Fédérale de Lausanne, 1015 Lausanne, Switzerland; 5 Laboratory for Microbiology, Gent University (UGent), 9000 Gent, Belgium

**Keywords:** Flo adhesin family, pathogenic yeasts, *Candida glabrata*, *Saccharomyces cerevisiae*, host-pathogen interaction, abiotic surface adhesion, adhesin structure, adhesin architecture

## Abstract

The first step in the infection of fungal pathogens in humans is the adhesion of the pathogen to host tissue cells or abiotic surfaces such as catheters and implants. One of the main players involved in this are the expressed cell wall adhesins. Here, we review the Flo adhesin family and their involvement in the adhesion of these yeasts during human infections. Firstly, we redefined the Flo adhesin family based on the domain architectures that are present in the Flo adhesins and their functions, and set up a new classification of Flo adhesins. Next, the structure, function, and adhesion mechanisms of the Flo adhesins whose structure has been solved are discussed in detail. Finally, we identified from Pfam database datamining yeasts that could express Flo adhesins and are encountered in human infections and their adhesin architectures. These yeasts are discussed in relation to their adhesion characteristics and involvement in infections.

## 1. Introduction

Fungal infections are an extremely important growing health problem since they kill over 1.6 million people worldwide per year [1,2,3]. Fungi are present everywhere in our environment and are, usually, harmless for healthy people. Fungal infections can be topical and local, such as surface infections on the skin or in the vaginal tract. Systemic infections arise when the fungi enter and proliferate in the bloodstream. Systemic fungal infections affect people with an altered immune system due to medical interventions, i.e., cancer therapy, organ transplantation, asthma and the use of immune-modulatory medications, immunosuppressive diseases (such as Acquired Immune Deficiency Syndrome (AIDS)) [4], or malnutrition (under- and overnutrition) [5,6,7]. In addition, viral pneumonia increases patients’ susceptibility to fungal superinfections. During the recent COVID-19 pandemic, COVID-19-associated pulmonary aspergillosis was responsible for a substantial increased mortality [8,9,10,11,12].

The contribution of fungal infections to the global burden of disease is largely unrecognised [1,13,14,15]. It is estimated that around 1.7 billion people have superficial fungal infections such as skin, hair and nail infections [1,15,16]. Mucosal fungal infections of the oral and genital tracts are also an extremely burden, especially vulvovaginal candidiasis. It was estimated that around 50 to 75% of women in their childbearing years suffer from at least one episode of vulvovaginitis, and 5 to 8% (around 75 million women) have at least four episodes annually [15,17]. Recent global estimates have found 3,000,000 cases of chronic pulmonary aspergillosis, ~223,100 cases of cryptococcal meningitis complicating HIV/AIDS, ~700,000 cases of invasive candidiasis, ~500,000 cases of *Pneumocystis jirovecii* pneumonia, ~250,000 cases of invasive aspergillosis, ~100,000 cases of disseminated histoplasmosis, over 10,000,000 cases of fungal asthma and ~1,000,000 cases of fungal keratitis occur annually [1]. Invasive fungal infections are of great concern because they are associated with unacceptable high mortality rates [15]. The epidemiology of invasive fungal infection is evolving [18,19,20]. A growing population of immunosuppressed patients has resulted in increasingly frequent diagnoses of invasive fungal infections, including those caused by unusual yeasts. The incidence of non-*albicans* species of *Candida* is increasing compared with that of *C. albicans*, and several species, such as *C. glabrata*, *C. lusitaniae*, *C. auris*, *C. inconspicua* and *C. krusei*, may be resistant to antifungal therapy.

Early accurate diagnosis allows prompt antifungal therapy; this is, however, often delayed or unavailable leading to high mortality rates, serious chronic illness or blindness [1]. The choice of available antifungal drugs to treat invasive fungal infections is limited, since only three structural classes of compounds are available, i.e., polyenes, azoles, and echinocandins [21]. Additionally, current antifungal drugs can show significant limitations, such as amphotericin B that displays a considerable toxicity and undesirable side effects [22,23], issues with pharmacokinetic properties and activity spectrum, a small number of targets [24,25], and they can interact with other drugs, such as chemotherapy agents and immunosuppressants [26,27]. Recently, there is an increased interest in the development of new antifungal compounds and multiple compounds are in clinical development stage [28,29,30,31,32,33,34].

Cell adhesion proteins are critical to fungal cell interactions in development, symbiosis, and pathogenesis [35]. They are specifically found on the outside of the cell wall [36]. They participate in mating, colony morphology changes, biofilm formation, fruiting body development, and interactions with mammalian and plant hosts. Many fungi contain a family of cell wall glycoproteins, called “adhesins” that confer them unique adhesion properties [37,38,39]. These molecules are required for the interactions of fungal cells with each other (flocculation, filamentation and biofilm formation) [37,38,40,41], inert surfaces such as agar and plastics [40,42,43,44] and host tissues [45,46]. Selective cell adhesion is also needed for fungal pathogenesis. The majority of these functionally characterized fungal adhesins are glycoproteins with a common architecture. A high-complexity cell surface exposed N-terminal adhesion domain for ligand recognition and binding. Followed by a large, low complexity domain characterized by a variable number of tandem repeats with significant intraspecies length polymorphisms and a C-terminal domain harbouring a glycosylphosphatidylinositol (GPI) anchor that mediates attachment to the glucan layer of cell walls [36,39,47,48,49].

Fungal cell wall adhesins are involved in the first step in pathogenesis, i.e., the adherence to host tissue or abiotic medical devices. This first step is critical for colonization leading to invasion and damage of host tissue or biofilm formation. Adherence of pathogenic fungi to host tissues can occur at different sites in the human body. In the case of epithelial and endothelial tissues, one of the potential adhesion targets is represented by the glycocalyx, i.e., the extracellular mesh of carbohydrate-rich molecules bound to the cell membranes or secreted by cells in the external medium [50]. The microbial adhesion to components of the glycocalyx, such as glycosylated host receptors or other glycoproteins, is often mediated by adhesion proteins endowed with lectin activity [51,52,53].

The most common yeast infection is candidiasis caused by *Candida* spp., while many other fungal species are also medically important [54]. *Candida* spp. can adhere to different surfaces such as skin and mucosal tissue as well as abiotic surfaces, an important step in establishment of infection [55,56]. Additionally, *Candida* cells are capable of ‘flocculating’ with other *Candida* cells as well as interacting with other microbes in the human microflora, forming large communities with reduced susceptibility to antifungals [57]. The most frequently encountered *Candida* species is *Candida albicans*; however, the incidence of non-*albicans* species, such as *C. glabrata*, *C. tropicalis*, *C. parapsilosis*, *C. intermedia*, *C. lusitaniae*, *C. haemuloni*, and *C. auris* has increased over recent decades due to the long-term use and limited options of antifungal drugs [58,59,60,61,62].

The Flo adhesin family was initially discovered in brewer’s yeast. Flo adhesins are involved for ages in ale (*Saccharomyces cerevisiae*) and lager (*S. pastorianus*) beer fermentation since cells “flocculate” (aggregate) at the end of the primary fermentation and the flocs sediment (lager beer) rapidly from the medium, or rise to the liquid surface and form a yeast layer [63]. Later, it was also found that Flo proteins are involved in processes where *S. cerevisiae* switch from a planktonic lifestyle to a complex multicellular structure such as—besides flocs—filaments, mats, flors, and biofilms in response to changes in the environment and its genetic background [64]. The potential of individual yeast cells to switch between different growth modes in nature is advantageous for optimal dissemination, protection, substrate colonization and escape unfavourable conditions at the population level [64,65,66,67]. Originally, the composition of the Flo adhesin family was based on the flocculation proteins/genes discovered in *S. cerevisiae*, i.e., Flo1p, Flo5p, Flo9p and Flo10p (and de transcription factor Flo8p) [37]. Later on, 2 subgroups were defined [38]. The members of the first subgroup are encoded by genes, including *FLO1*, *FLO5*, *FLO9*, and *FLO10*, which share considerable sequence homology. The gene products of *FLO1*, *FLO5*, *FLO9*, and—to a lesser extent—*FLO10* [44] promote cell-cell adhesion and contribute to the formation of multicellular clumps (flocs), and, therefore, these adhesins were called flocculins [68]. The members of the second group of the Flo family, including Flo11p, Fig2p, and Aga1p, have a domain structure such as that of the first, but with quite unrelated amino acid sequences. Flo11p also promotes cell-cell adhesion, but does this only weakly [44,69]. Flo11p is mainly required for diploid pseudohyphal formation, haploid invasive growth [40,70], mat [71] and biofilm formation [72,73]. N-Flo11p does not bind mannose, which contrasts with the other Flo proteins. However, N-Flo11p can interact with N-Flo11p (homophilic adhesion ability), explaining the weak-flocculation characteristic [74,75]. Fig2p and Aga1p are induced during mating [76,77]. Aga1p, linked by disulphides to the soluble peptide, Aga2p [78], is required on the surface of *MATα* cells for them to adhere to the protein Sag1p on the surface of *MAT*α cells [79].

In this review, we redefine the Flo adhesin family based on the protein architecture of the Flo proteins *sensu stricto*. Based on this new definition, we reviewed the adhesins containing these Flo protein architectures that were found to be present in yeasts that have been isolated from human infections. We discuss the structure, function, and binding mechanisms of members of the Flo adhesin family of which the protein structure has been solved. Next, we review and discuss the yeasts that express Flo protein type adhesins.

## 2. Redefinition of the Flo Adhesin Family Based on the Protein Architecture

The Flo adhesin Family can be redefined based on the domain architectures present in Flo adhesins since these domains will define the functional properties of the adhesins. The Flo family is composed of 2 flocculation adhesin classes, i.e., the Flo-type and the Flo11-type adhesins. The Flo type can be further divided into the Flo adhesins that contain a PA14 or GLEYA lectin domain and a flocculin domain (P00624) and/or a flocculin type 3 repeat (flocculin_t3) (PF13928) belong to the lectin type flocculins. The N-terminal PA14/GLEYA domain is the essential domain since it contributes most to the adhesion strength via its lectin function.

**Figure 1:**
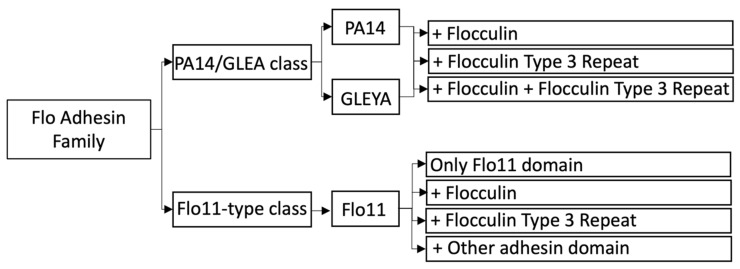
Definition of the Flo adhesin family based on the structural architectures of the Flo adhesins.

**Figure 2:**
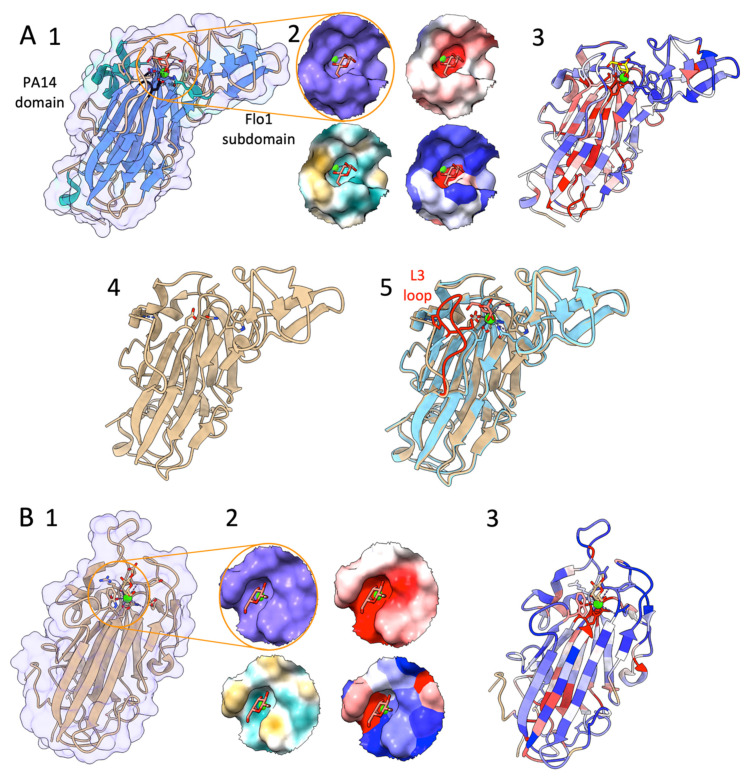
(**A**) **1**. Structure of the N-terminal part of Flo1p (from PDB entry 4LHN). The “D*cis*D” motif is indicated in black by the residues Asp160 and Asp161. **2**. Mannose-binding pocket surface zoomed view (top left), electrostatic surface (top right), hydrophobic (brown)-hydrophilic (cyan blue) surface (bottom left), conserved amino acids coloured surface (bottom right). **3**. Colouring of the structure by sequence conservation; low to high conservation: from blue (−1.8) to white to red (1.9) (calculated via the ConSurf server [105,106]). **4**. The apo structure (from PDB entry 4LHL). **5**. Projection of the conformations containing the mannose ligand (blue coloured; PDB 4LHN) to the apo conformation (blown coloured; PDB 4LHN). Loop L3 (red coloured) closes upon mannose binding. (**B**) **1**. Structure of N-Epa1p (from PDB entry 4A3X). **2**. Galactose-binding pocket surface zoomed view (top left), electrostatic surface (top right), hydrophobic (brown)-hydrophilic (cyan blue) surface (bottom left), conserved amino acids coloured surface (bottom right). **3**. Colouring of the structure by sequence conservation; low to high conservation: from blue (−1.4) to white to red (2.1) (calculated via the ConSurf server [105,106]).

The Flo11 type adhesins can be subdivided into architectures containing only the Flo11 domain, the Flo 11 domain and the flocculin domain or the flocculin type 3 repeat, and the Flo 11 domain and another adhesin structural domain (Figure 1). Based on this definition of the Flo adhesin family, Fig2 and Aga1p do not belong anymore to this family since Fig2p (Pfam: FIG2_YEAST, P25653) does not contain a Flo11 domain (nor a PA14 or GLEYA domain); it does contain only a Flocculin_t3 domain. Aga1p (Pfam: AGA1_YEAST, P32323) does not contain any of the specified domains for flocculation adhesins.

The flocculin repeat domains (P00624) were initially found in the Ser/Thr-rich central region of Flo1p, Lg-Flo1p, Flo5p, Flo9p and Flo10p and correspond to the tandem repeats, which are important for proper cell wall targeting and presentation of the adhesins [37,49,80]. The Ser and Thr amino acids are prone to extensive O-glycosylation during post-translational modification and enable the adhesins to attain a long, semi-rigid rod-like structure [81]. An increasing number of tandem repeats increases the strength of the adhesion [82,83,84]. The repeats trigger frequent recombination events within the gene or between the gene and a pseudogene, resulting in expansion and contraction in the gene size, which affects the adhesion properties of the cells [82].

The flocculin type 3 repeat (PF13928) was initially found in Flo5p, Flo9p, and Lg-Flo1p close to the C-terminus of the adhesin. The presence of these domains on the functional characteristics of the adhesin and on the cell adhesion properties has not yet been investigated.

## 3. Structure and Function of Flocculation Adhesins

The member proteins of the adhesin family have a modular configuration that consists of three domains (N-terminal, central and C-terminal domain) and an amino-terminal secretory sequence that must be removed when the protein moves to the plasma membrane through the secretory pathway [35,49,85,86]. The GPI anchor is modified as the proteins become linked to β-1,6-glucan in the wall. Despite the intensive research on yeast adhesion, a relative low number of adhesin structures have been investigated at the molecular level and their structure solved [86] (Table 1).

### 3.1. PA14/GLEYA Flo Type Adhesin Structure

The adhesins that belong to this type, contain a PA14 domain (Pfam family PA14, PF07691) or a GLEYA domain (Pfam family GLEYA, PF10528) in the N-terminal part of the adhesin. The PA14 domain family was discovered based on the sequence analysis of an insert in bacterial β-glucosidases, which was also found in other glycosidases, glycosyltransferases, proteases, amidases, yeast adhesins, and bacterial toxins [87]. The insert is a 14-kDa region of PA_20_, which is a fragment of the protective antigen (PA) from anthrax toxin, has a β-barrel structure [88]. The PA14 domain is present in 2448 species, 974 protein architectures, and in 54 solved protein structures (Pfam 34.0, March 2021). The presence of a calcium-dependent carbohydrate-binding pocket is a common element in the PA14 domain family [89,90]. The GLEYA domain is structurally related to lectin-like binding domains found in fungal adhesins such as the *S. cerevisiae* Flo proteins and the *C. glabrata* Epa proteins [91]. The distinction is not always clear as can be noted from the Uniprot description of the adhesins containing a GLEYA domain (Table 1). An EYDGA pentapeptide motif belonging to the PA14 domain was identified [92] and was found to be present in the N-terminal domain of Epa1 from *C. glabrata*, where it is involved in carbohydrate binding. This motif is comparable to the VSWGT pentapeptide in Flo1p from *S. cerevisiae* [91]. The VSWGT motif of Flo1p and the EYDGA motif are present in the same position within a hypervariable region of the PA14 domain [93]. The VSWGT/KVLAR motif of Flo1p/Lg-Flo1p and the EYDGA motif of Epa1p represent a surface loop between two β-strands, 9 and 10, in the structure of the anthrax toxin PA domain [88]. Adhesins with a GLEYA domain possess a typical N-terminal signal peptide and a domain of conserved sequence repeats but lack GPI anchor attachment signals [91]. However, it was demonstrated for Epa1 that the GPI anchor is essential both for cross-linking in the cell wall and for Epa1-mediated adherence [45,94]. The GLEYA domain contains a conserved motif G(M/L)(E/A/N/Q)YA, hence the name GLEYA. Based on sequence homology, it was suggested that the GLEYA domain would predominantly contain β-sheets, which was later confirmed by the solved structures of Epa1p and Epa9p (Table 1) [92,95].

Several of the N-terminal adhesion domains of the PA14 type Flo proteins were solved (Table 1), i.e., N-Flo5p [100] and N-Flo1p from *S. cerevisiae* [93] (Figure 2A), and N-Lg-Flo1p from *S. pastorianus* [93,96]. The atomic structures of N-Flo1p, N-Lg-Flo1p, N-Flo5p, N-Epa1p, N-Epa6p, and N-Epa9p are very similar (Figure 2). The main body of these proteins, i.e., the PA14/GLEYA domain, is a β-sandwich fold made up of two antiparallel β-sheets and an L-shaped region composed of the N and C-terminal regions (Figure 2A,B). N-Flo1p and N-Flo5p contain a protruding β-sheet subdomain (the Flo1/Flo5 subdomain) that is located at one end of the protein, close to the carbohydrate binding site (Figure 2A1). In Lg-Flo1p and N-Epa1, this subdomain is replaced by a short highly flexible loop 2 [95,96,99]. The high flexible loop 3 (L3) is present in N-Flo1p and N-Lg-Flo1 (Figure 2A), as well as in N-Flo5p and N-Epa1 (Figure 2B); and has a significant effect on carbohydrate recognition. In contrast to N-Flo5p, this loop of N-Flo1p is closer to the binding side and lysine 194 (K194) from this loop interacts directly with the carbohydrate, which results in a three-fold increase in affinity for mannose compared to N-Flo5p. In Epa1p, the L3 loop establishes stronger stacking interactions with the ligands galactose and galactose-terminating glycans via tryptophan 194 (W194) (which corresponds to K194 in Flo1p) [92,95]. The carbohydrate-binding pocket of N-Lg-Flo1p is more enclosed than the one of N-Flo1p, which results in a 10 times higher affinity for the ligand mannose [93]. Mannose disaccharides and high-mannose glycans fit differently in the binding sites of N-Flo1p and N-Flo5p, which results in a different specificities and affinities. Longer mannose-containing oligosaccharides do not interact well with N-Lg-Flo1p due to the steric hindrance encountered in the binding site.

The binding site of these proteins contains a calcium ion that is directly involved in carbohydrate binding (Figure 2). In N-Flo1p and N-Flo5p, Ca^2+^ is coordinated on carbohydrate binding loop 1 by *cis* peptides aspartic acid 160 (D160) and D161 (indicated as “D*cis*D” motif) (Figure 2A1), and on CBL2 by the asparagine 224 (N224) side chain and the carbonyl groups of valine 226 (V226) and W228. These residues are strongly conserved in the Flo and Epa adhesin families due to their importance for metal binding (Figure 2A3,B3) [95,100].

Flocculation cell-cell binding in floc or biofilm formation is based on the lectin function of the PA14/GLEYA Flo type adhesins. N-Flo1p and N-Flo5p binds specifically to D-mannose glycans [93,100,101]. The affinity for these lectins is around 10 times larger than for monosaccharides [86]. N-Lg-Flo1p displays a broader specificity towards sugars [93,96]. Expressed PA14/GLEYA Flo type adhesins are the dominant cell wall proteins that stick out of the cell wall [102]. On flocculating cells, N-Flo1p interacts homophilically with the glycans of N-Flo1p of the interacting cell in the presence of Ca^2+^ [93]. In addition, it was demonstrated that glycan-glycan interactions with the involvement of Ca^2+^ interactions contribute to cell-cell interactions [93], and that these interactions are likely involved in the first intercellular contacts [103,104]. These results pointed to a two-step cell-cell adhesion mechanism, where in the first step the long, flexible glycans have a high probability of interaction when the cells are moving close to each other and initially serve to stabilize cell-cell interactions. In the next step, the non-reducing glycan end enter the binding pocket of the lectin and binds to the protein. In both steps, Ca^2+^ is crucial for the interactions.

It has been recently discovered that amyloid-like bonds can contribute to *C. albicans* cell-cell interactions via the Als adhesins [107,108,109]. These intercellular bonds show properties of cross-β aggregation and in addition to the interactions that cluster the adhesins on yeast cell surfaces [110]. Data on Flo1p also support the formation of cross-β bonds in *trans* between expressing cells [109]. The N-Flo1p domain is followed by a variable number of tandem repeats that are predicted to have anti-parallel β-sheet structure, and these repeats unfold under extension or shear force [110,111].

### 3.2. Flo11 Type Adhesin Structure

The expression of the *S. cerevisiae* flocculation protein Flo11p can play a role in lifestyles involving complex multicellular structures such as flocs, filaments, mats, and flors a major role in these lifestyles, which give yeast selective advantages to survive in specific growth conditions [40,64,70,112,113]. When the carbon source (e.g., glucose) is depleted in the growth medium, *FLO11* is expressed, which makes haploid *S. cerevisiae* cells adherent and allows them to invade into semi-solid agar medium; this is called “invasive growth” [44,66,114]. Diploid cells will adopt—when nitrogen becomes limiting in the growth medium—an elongated shape and form filaments that grow from the colony edge; this is called diploid pseudohyphal growth [44,115,116,117]. Flo11p can also be involved in the formation of mats, which are complex colony-like structures on a low-density (0.3%) semi-solid medium (that resembles the environment of rotting fruit on which these yeasts can grow [118]) [43,71,119,120]; the formation of a flor (or velum), which is the air-liquid interfacial cellular aggregation in the process of wine (beer) fermentations [121,122,123,124]. The adherence of cells to solid surfaces (such as glass, stainless steel, agar, and plastics) can also lead to the development of biofilms [43,44,125,126]. Cell–cell interaction (floc formation) can also be based on Flo11p interaction [44,49,69,72,74,75,112,127]. Many parameters influence the expression of *FLO11* and flocculation activity such as the cell density, surface charge, and pH, and environmental factors such as oxygen limitation, nutrient limitation, and cell surface hydrophobicity [127,128,129,130]. Flo11p mediates different processes in different strains [38,66,72,74,112,127], and strain-specific differences in the level of flocculation result from significant sequence differences in the *FLO11* alleles, and do not depend on quantitative differences in *FLO11* expression or surface hydrophobicity [131].

**Figure 3:**
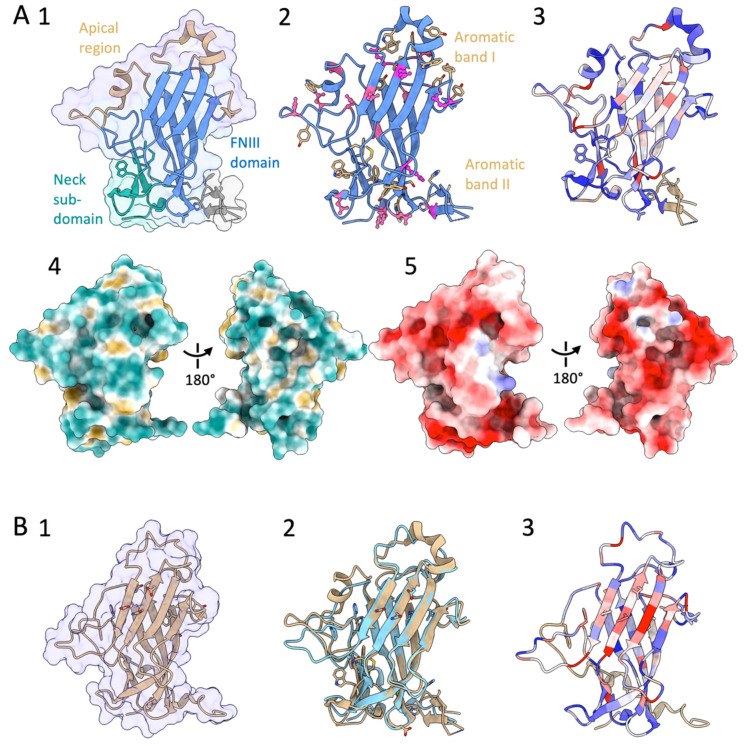
(**A**) **1**. Structure of the N-terminal part of *S. cerevisiae* Flo11p (N-ScFlo11p) (from PDB entry 4UYR). **2**. Indication of the aromatic residues Trp and Tyr (in brown), and the acid residues Asp (in pink) and Glu (in magenta). **3**. Colouring of the structure by sequence conservation; low to high conservation: from blue (−1.6) to white to red (1.8) (calculated via the ConSurf server [105,106]). **4**. Hydrophobic (brown)-hydrophilic (cyan blue) surface (PDB 4UYT), and **5**. electrostatic surface (PDB 4UYR). (**B**) **1**. Structure of the N-terminal part of *K. pastoris* Flo11p (N-KpFlo11p) (from PDB entry 5FV5). **2**. Matching the conformation of N-KpFlo11p (PDB 5FV5) (blue) to the one of N-ScFlo11p (PDB 4UYR) (brown). **3**. Colouring of the structure by sequence conservation; low to high conservation: from blue (−1.6) to white to red (2.3) (calculated via the ConSurf server [105,106]).

The structures of two N-terminal adhesion domains of Flo11p have been solved by using X-ray crystallography (Table 1), i.e., the one of Flo11p from *S. cerevisiae* (N-ScFlo11p) [69] and recently the one from *Komagataella pastoris* (N-KpFlo11p) [99]. Despite a sequence identity between-N-KpFlo11p and N-ScFlo11p of only 32%, their overall structures showed a high degree of similarity after superposition [99] (Figure 3B2). Three subdomains can be distinguished: a hydrophobic apical region, a β sandwich of the fibronectin type III domain (FN3-like domain), and the neck subdomain (Figure 3A1,B1). The core domain is the β sandwich that is formed by the antiparallel β sheets I and II and was assigned to the class of fibronectin type-III-like domains (FNIII). This core domain showed the highest degree of similarity between the two N-Flo1p adhesin domains [99], and this domain is well conserved (Figure 3A3,B3). The FNIII fold forms a large family within the immunoglobulin (Ig) superfamily that includes cell adhesion proteins, cell surface hormone and cytokine receptors, chaperones, and carbohydrate-binding domains [132]. The FNIII-like domain subtype shows a seven-stranded strand-switched type, where sheet I consist of three strands and sheet II of four strands (Figure 3A1,B1). The FNIII fold differs from other Ig folds by its fourth strand, which is part of the second, but not the first, β sheet [69].

The FNIII-like domain contains by two surface aromatic bands at the apical region and the neck subdomain (Figure 3A2) [69,99]. These aromatic bands are well conserved (Figure 3A3,B3). Hydrophobic interactions between these aromatic surface features, whose propensity for interaction is ameliorated in a pH-dependent manner by co-distributed acidic residues (Figure 3A2,A5), mostly determine the homophilic recognition by the Flo11 adhesin domains (Figure 3A4). Even though these hydrophobic interactions are less specific than the lectin–carbohydrate interactions of the other Flo adhesins, they can excel by their long range of attractive forces. Single-cell force spectroscopy showed that these N-Flo11p domains confer remarkably strong adhesion forces between cells, leading to efficient cell aggregation and biofilm formation [99]. The co-alignment of Flo11 fibres from opposing yeast cells could be observed by scanning electron microscopy, indicating that Flo11p acts as a spacer-like, pH-sensitive adhesin that resembles a membrane-tethered hydrophobin [69].

As for Flo1p, data on Flo11p also support the involvement of this adhesin in the formation of cross-β bonds *in trans* between cells [109]. There are potential amyloid core sequences in the post N-terminal domain and C-terminal regions [133]. As for Flo1 and Als adhesins, the ability to form cellular aggregates can be induced by shear force.

## 4. Yeasts Expressing Flo Proteins Involved in Human Infections

### 4.1. Pathogenic Candida Species

#### 4.1.1. *Candida glabrata*

*C. glabrata* strains were originally classified in the genus *Cryptococcus* and next *Torulopsis* due to its lack of filaments formation, and was in 1978 classified in the genus *Candida* due to it human pathogenicity [134,135]. *C. glabrata* is more closely related to *S. cerevisiae* than to *C. albicans* [59,134,136,137,138,139]. It is a major opportunistic human fungal pathogen that has become the second most frequent cause of *Candida* infections [134,140,141,142,143]. It is a nondimorphic yeast that exist as small blastoconidia under all environmental conditions as a pathogen (it does not form pseudohyphae at temperatures above 37 °C) [134]. *C. glabrata* can cause superficial and life-threatening dissemination infections reaching high mortalities of around 40% [144]. Immunocompromised, cancer and diabetic patients are particularly susceptible [18,54,142,145,146,147]. *C. glabrata* shows a high antifungal resistance against azole antifungal agents [24]. It can adhere to host tissues cells as well as to abiotic surfaces and colonize them as biofilms, which further increase the antifungal resistance and evade the host immune defences [144,148,149,150,151,152]. Biofilms on medical devices (e.g., indwelling catheters or prosthetic heart valves) can result in failure of the device and the cells in the biofilm can initiate future continuing infections [153,154,155,156,157].

*C. glabrata* can express many adhesin-encoding genes and genome comparisons with closely related species, including *S. cerevisiae*, revealed a correlation between the number of adhesin-encoding genes and pathogenicity [152,158,159]. The adhesins from the Epa (“epithelial adhesion”) family are up-to-now the best characterised adhesins from *C. glabrata*; the structures of N-Epa1p [92,95,98], N-Epa6p [98], and N-Epa9p [98] have been solved recently [86]. These N-terminal Epa adhesin domains contain a GLEYA domain with lectin activity, which is Ca^2+^ dependent, and recognizes a wide variety of glycans with terminal galactose residues linked via α- or β-glycosidic bonds to a secondary sugar for conferring epithelial cell adhesion [53,98] *C. glabrata* can also express Epa23p, which can be classified as a PA14/GLEYA-type flocculin since the adhesin architecture is composed of a PA14 domain and 5 flocculin repeat domains (Table 2). In the other members of the Epa family, such as Epa1p, Epa2p, Epa3p, Epa6p and Epa 9p, only the GLEYA domain is present in the N-terminal region of the adhesin.

Epa adhesins mediate adherence to human epithelial and endothelial cells by recognizing glycans containing terminal galactose residues [47] and show the highest affinity for the Thomsen-Friedenreich (T or TF) antigen (Galβ-1,3-GalNAc), which likely mediates N-Epa-p adherence to highly glycosylated proteins such as mucins [95]. It was also demonstrated that N-Epa1p binds to fibronectin from human plasma [53,160] as well as to mucin [53]. Many other human receptors of Epa adhesins have been predicted by constructing their lectin-glycan interaction (LGI) network, which is an approach that is based on linking glycan array screening results of the adhesins to a human glycoprotein database [53]. The Epa1p, Epa6p and Epa7p LGI network revealed that a large set of receptors are present in body fluids or displayed on human cells in various body systems; receptors included several mucins (urogetinal, respiratory, exocrine and digestive system), κ-casein (exocrine system), epidermal growth factor receptor (EGFR, urogenital system), cadherin-5 (CD144, urogenital system), lactotransferrin (LTF, exocrine system), serotransferrin (TF, hemic system), immunoglobulin M (IgM, hemic system), tumor necrosis factor alpha (TNF-α, hemic system), P-selectin glycoprotein ligand (PSGL-1, hemic system), leukosialin (CD43, respiratory system), receptor-type tyrosine-protein phosphatase C (CD45, hemic system), von Willebrand factor (vWF, hemic system), β-secretase 1 (BACE1, urogenital system), lysosome-associated membrane glycoprotein ½ (LAMP1/2, hemic system), plasminogen (PLG, urogenital system).

#### 4.1.2. *Candida lusitaniae* (*Clavispora lusitaniae*)

*Clavispora lusitaniae*, which is the teleomorph of *C. lusitaniae*, is an environmental saprophytic yeast that belongs to the CTG clade of *Candida* [161] (Table 2). *C. lusitaniae* can behave rarely as an opportunistic pathogen in humans, and its most common risk factors include immunocompromised patients often with an underlying malignancy [162]. However, nosocomial acquisition secondary to an indwelling urinary catheter [163], and catheter-associated *C. lusitaniae* candidemia [164] have also been reported. Amphotericin B resistance among isolates of *C. lusitaniae* has distinguished it among *Candida* species [165]. The adhesion of *C. lusitaniae* to abiotic surfaces (polystyrene and steel surfaces) has been demonstrated [166].

*C. lusitaniae* strains can express adhesins that contain an N-terminal GLEYA domain and a flocculin repeat domain (Table 2), as well as adhesins that contain one or more N-terminal Flo11 domains and none or multiple flocculin type 3 repeats (Table 2). It is expected that multiple Flo11 adhesin domains will increase the interaction strength. Currently, these adhesins have not been characterized at the molecular nor cellular level, and very little is known about their role in the adhesion process. Recently, it was shown that when the Flo11 domain from *C. lusitaniae* was expressed in an *S. cerevisiae* expression system, adhesive growth was conferred to *S. cerevisiae* [99].

#### 4.1.3. *Candida parapsilosis* and *Candida tropicalis*

*C. parapsilosis* can be isolated from domestic animals, insects, the soil and marine environments [167,168]. *C. parapsilosis* (*sensu lato*) was reclassified as a fungal complex formed by three genotypically distinguishable species, i.e., *C. parapsilosis* (*sensu stricto*), *C. orthopsilosis* and *C. metapsilosis* [169]. The opportunistic yeast *C. parapsilosis* is responsible for 20–30% of all fungal infections, particularly those related to the usage of catheter and other medical devices, and it became the second most frequently opportunistic yeast isolated from bloodstream infections in different clinical settings around the world, especially in Latin America and Asia [170,171,172,173,174,175,176,177,178,179,180,181,182]. *C. parapsilosis* is associated with a pronounced capacity to adhere to plastic surfaces and several other implanted devices, and consequently to the development of candidaemia related to catheters [153,183]. The adhesion of *C. parapsilosis* to polystyrene and steel surfaces has been demonstrated [166]. Pseudohyphal formation was also positively correlated with adhesion of *C. parapsilosis* complex strains abiotic surfaces, such as polystyrene and glass [183], and acrylic surfaces [184]. Cell surface hydrophobicity can have a significant effect on the initial events leading to adherence [185] and it has been shown that it can make *C.* *albicans* and *C. glabrata* more virulent [186]. An elevated cell hydrophobicity has been recorded for clinical *C. parapsilosis* complex strains [183,187].

*C. tropicalis* is a diploid dimorphic yeast, which lives either as budding cells or as a pseudomycelium; in rare cases it can form true hyphae (as for *C. albicans*) [188,189]. It is taxonomically close to *C. albicans* and shares many pathogenic traits [190]. It is one of the three most commonly isolated non-*albicans Candida* species [168,191,192,193,194,195,196,197]. It is mainly isolated from blood and urine samples [192,198,199]. It is also often detected in patients requiring prolonged catherization, receiving broad-spectrum antibiotics and with cancer [195,198,200,201,202]. In addition, *C. tropicalis* displayed a higher potential for dissemination in neurotropenic individuals compared to *C. albicans* and other non-*albicans Candida* species [195].

A bioinformatic search of pathogen-specific gene families of *Candida* species (*C. parapsilosis*, *C. tropicalis*, *L. elongispora*, *C. guilliemondii*) revealed several genes for putative cell wall adhesins-like proteins such as Als-like adhesins, Hyr/Iff proteins, and Pga30-like proteins (predicted glycosylphosphatidyl-inositol-anchored protein 30) [203,204]. Genome data also allowed to identify five genes homologous to the *ALS* (agglutinin-like sequence) gene family of *C. albicans* in *C. parapsilosis*, three in *C. orthopsilosis* and four in *C. metapsilosis* [205]. Considerable variation was noted in relative gene expression for isolates of the same species. It was shown that the gene *CpALS7* showed a positive correlation with adhesion to extracellular matrix proteins under fluid shear stress [206]. Site-specific deletion of *CpALS7* reduced the adhesion of *C. parapsilosis* to buccal epithelial cells and significantly attenuated virulence in a murine model of urinary tract infection [207].

One *C. parapsilosis* Flo11-type adhesin and one in *C. tropicalis* were found in the Pfam database (Table 3). They both have the same protein architectures since besides the Flo11 domain they contain also two flocculin type 3 repeats. Their molecular characterization, virulence, and role of these adhesins in the adhesion process is currently not yet known and needs further investigation. These Flo11-type adhesions could play a role in biofilm formation since it was shown that *C. parapsilosis* and *C. tropicalis* displayed a high biofilm formation ability as well as a high adhesion ability [208].

#### 4.1.4. *Candida auris*

*C. auris* was first described in 2009 in Japan [209]. It is an emerging multidrug-resistant fungal pathogen of the *Candida/Clavispora* clade that is becoming a worldwide public health treat over the past decade due severe invasive infections with high mortality rates [210,211,212,213,214]. Due to its capability of nosocomial transmission and forming adherent biofilms on clinically important substrates, a high number of related hospital outbreaks have been reported worldwide [215]. Recently, genomic analyses revealed that early contemporaneously detected cases of *C. auris* were geographically stratified into four major clades [216]. While Clades I, III, and IV are responsible for ongoing outbreaks of invasive and multidrug-resistant infections, Clade II, also termed the East Asian clade, consists primarily of cases of ear infection, is often susceptible to all antifungal drugs, and has not been associated with outbreaks.

The virulence factors associated with *C. auris* infections are not yet completely understood [217]. *C. auris* pathogenic attributes that have been identified include pathways required for cell wall modelling and nutrient acquisition, two-component systems, the production of hydrolytic enzymes such as phospholipases and proteinases that are likely involved in the adherence and invasion of host cells and tissues during infections, other mechanisms of tissue invasion, and immune evasion and multidrug efflux systems [217,218,219,220,221,222,223]. Other adhesin genes identified in *C. auris* include orthologs of *C. albicans ALS* genes such as *ALS3* and *ALS4*, while Als3p was identified on *C. auris* cell surface by anti-*C. albicans* Als3p antibodies [218,224]. Subtelomeric dynamics and the conservation of cell surface proteins (including Hyr/Iff-like and novel candidate cell wall proteins, and an Als-like adhesin) in the clades responsible for global outbreaks causing invasive infections suggest an explanation for the different phenotypes observed between clades [216].

*C. auris* can form biofilms on several indwelling medical devices, such as catheters, central/peripheral line tips, and neurological shunts [223,225,226]. Biofilm formation protects *C. auris* from triazoles, polyenes, and echinocandins antifungal drugs [227,228]. It was shown. That seven adhesin genes (*IFF4*, *CSA1*, *PGA26*, *PGA52*, PGA7, *HYR3* and *ALS5*) were upregulated during biofilm formation [227]. The GPI-anchored cell wall genes (*IFF4*, *CSA1*, *PGA26*, *PGA52*) were upregulated at all time points during in vitro biofilm formation, while *HYR3* and *ALS5* were only upregulated in mature biofilms [227,229]. Additionally, key role genes involved in biofilm extracellular matrix formation, such as encoding efflux pumps (*MDR* and *CDR* homologs) and glucan-modifying enzymes, were upregulated during biofilm formation, and their inhibition improved the susceptibility of biofilms to fluconazole [228,229,230].

We found one Flo11 type adhesin in the Pfam database (Table 3). In addition to the N-terminal Flo11 domain, it contains a collagen triple helix repeat (Collagen (PF01391)) in the middle–C-terminal region of the protein. The collagen triple helix or type-2 helix is the primary secondary structure of various types of fibrous collagen, including type I collagen [231,232]. It consists of a triple helix made of the repetitious amino acid sequence glycine-X-Y, where X and Y are frequently proline or hydroxyproline. This Collagen domain could mechanically stabilize the adhesin allowing it to stick out as a straight rod from the cell surface reaching for receptors/surfaces to interact with. As Flo11p in *S. cerevisiae* is involved in pseudohyphal growth, one suggestion is that this adhesin also plays a role in pseudohyphal-like aggregate formation in *C. auris*. These aggregates of pseudohyphal-like cells cannot be disrupted physically or chemically with detergents [223]. The ability to aggregate was shown to be an inducible trait since aggregate formation was stimulated by the prior exposure of *C. auris* to triazoles or echinocandins [233]. Aggregative phenotypes have been predominantly isolated from colonized patients and have higher capacity for biofilm formation than non-aggregative phenotypes [234,235]. A study showed that aggregate formation may help *C. auris* to evade immune recognition and thus facilitate its persistence in tissues [225]. This contrasted with another study where mice survived a high-dose *C. auris* intravenous challenge, even after cyclophosphamide -induced immunosuppression, in C5 complement deficiency in A/J mice and mice deficient in neutrophil elastase [236]. These contrasting results are likely due to differences in virulence of the tested strains and/or the infection model [217].

#### 4.1.5. *Candida* Species That Rarely Cause Infections

*Meyerozyma guilliermondii* (anamorph *C. guilliermondii*) is an ascomycetous yeast, which is widely distributed in nature, the human skin and the mucosal microflora [237,238]. Isolates identified as *C. guilliermondii* (teleomorph *Pichia guilliermondii*) were included in the new *Meyerozyma* genus by Kurtzman and Suzuki in 2010 [239]. The *M. guilliermondii* complex is a genetically heterogeneous complex comprising several phenotypically indistinguishable taxa, including *M. guilliermondii*, *C. fermentati*, *C. carpophila*, and *C. xestobi* [240,241,242]. With several unique characteristics and physiology, such as the wide substrates spectrum and capability of various chemicals synthesis, *M. guilliermondii* has been recognized for its biotechnological applications such as industrial enzyme production, metabolites synthesis and biocontrol capacity [243]. The incidence of human infections is low (ranges from 1 to 3% depending on the geographic region [244,245,246]), but cases of candidemia (especially in patients with cancer), endocarditis and invasive disease have been reported and increased over the years [237,246,247,248,249,250,251,252,253,254,255,256,257,258,259,260,261,262,263]. Despite the low incidence of candidaemia caused by this organism, *M. guilliermondii* is of particular clinical significance as it exhibits increased resistance to antifungal agents (azoles and echinocandins), compared to other *Candida* species [244,264].

We found two Flo11-type *M. guilliermondii* adhesins in the Pfam database: in one strain the protein contained only the Flo11 domain whereas in the other strain an additional flocculin type 3 repeat was present. These Flo11-type adhesins could play a role in surface adhesion and biofilm formation since it was demonstrated that *M. guilliermondii* has a high adhesion ability (comparable to the ones of *C. tropicalis* and *C. parapsilosis*) as well as a high biofilm formation ability [208]. Recently, the N-terminal region (containing the Flo11 domain) of a Flo11-type adhesin from *M. guilliermondii* was introduced into an *S. cerevisiae* expression system based on the *S. cerevisiae* Flo11p and allowing the presentation of the adhesin domain at the cell surface for functional analysis [99]. It was shown that the Flo11 domain from *M. guilliermondii* was competent to confer adhesive growth. In addition, the expression of the Flo11 N-terminal domains from *C. lusitaniae* (see above), *S. paradoxus*, *Kluyveromyces lactis*, *Torulospora delbrueckii*, and *Komagataella pastoris* were expressed and conferred also adhesive growth to *S. cerevisiae*, which indicates that the capacity of the Flo11 domains for conferring cellular adhesion is highly conserved in Saccharomycetales.

*C. intermedia* is rarely reported as a human pathogen. Catheter-related fungemia caused by *C. intermedia*, which were treated successfully with fluconazole and catheter removal, have been reported [265]. Misidentification of *C. duobushaemulonii* (which is also a human pathogen) as *C. intermedia* has also been reported recently [266]. We found two Flo11-type adhesins in the Pfam database: one that contains only one Flo11 domain and one that contains six Flo11 domains in the N-terminal region of the protein as well as five flocculin type 3 repeats at the C-terminal region (Table 3). These adhesions could play a role in catheter adhesion and biofilm formation.

*C. viswanathii* was isolated from cerebrospinal fluid (CSF) of a fatal case of meningitis, and was reported as a new yeast species by Viswanathan and Randhawa in 1959 [267]. Later, the yeast was also found in routine sputum cultures and a detailed description of the fungus including the Latin diagnosis was provided, and its taxonomic nomenclature validated [268]. A recent study evaluating the pathogenicity for normal and cortisone-treated mice showed that *C. viswanathii* is an opportunistic pathogen [269]. Due to a lack of mycological expertise for comprehensive phenotypic characterization in a vast majority of laboratory diagnostic centres, the prevalence of *C. viswanathii* in clinical and environmental samples is currently likely underestimated. We identified one Flo11-type adhesin that contained only one Flo11 domain in the N-terminal region, in the Pfam database.

*C. fabianii* (teleomorph *Cyberlindnera fabianii*) is an ascomycetous yeast of the *Phaffomycetaceae* family. It has been described under the genus *Hansenula*, *Pichia* and *Lindnera* [270], and next as *Cyberlindnera* along with 20 other taxa since the genus *Lindnera* was a later homonym of an already published genus *Lindnera* in 1866 [271]. *C. fabianii* rarely been reported as a human pathogen, but due to advanced diagnostic methods and therapeutic techniques, infection has been increasingly recognised [272,273,274,275,276,277,278,279,280,281,282,283,284,285,286,287,288]. One Flo11-type adhesin that contains two Flo11 domains in the N-terminal region, was found in the Pfam database (Table 3).

*C. haemulonii* is a rare *Candida* subtype that is an emerging and virulent yeast pathogen. *C. haemuloni* infection have been wide spread, ranging from South America, Asia, the Middle East and Europe [289]. The first case report of *C. haemulonii* infection in the United States was in 1991 [289], a second in 2020 [62]. Species identification is difficult due to phenotypic similarity to other *Candida* subtypes, such that there is a high risk of inappropriate antimicrobial administration and worsening of emerging resistance patterns. *C. haemulonii* has a proclivity for infection of chronic lower extremity wounds particularly in diabetic patients [62]. One Flo11-type adhesin that contains one Flo11 domains in the N-terminal region and collagen triple helix repeat, was found in the Pfam database (Table 3).

*C. inconspicua* was firstly described as *Torulopsis inconspicua* and later reclassified in *Candida* [290]. The species belongs to the *Pichia cactophila* clade, together with *P. kudriavzevii* (synonym *C. krusei* [291]), *Pichia norvegensis*, *P. cactophila*, and *Pichia pseudocactophila* [292,293]. *C. inconspicua* is genetically similar and phenotypically identical to *P. cactophila* and it has been suggested that they represent different sexual stages of the same species [270,294]. Genome sequencing of the type strain (CBS180) and several clinical isolates uncovered the hybrid origin of *C. inconspicua* [292]. *C. inconspicua* is a an emerging pathogen responsible for infections that are more prominent in European countries [294,295,296]. Most of the infections are associated with osteomyelitis, oropharyngeal and esophageal candidiasis in HIV positive patients, as well as with candidemia in patients with hematological malignancies [295,297,298]. *C. inconspicua* showed a low susceptibility to fluconazole and other antifungals [291,292,293,294,295,296,297,298,299,300,301]. One Flo11-type adhesin that contains one Flo11 domains in the N-terminal region and three CMB_1 (Carbohydrate-binding module, fungal cellulose binding domain) domains, was found in the Pfam database (Table 3). In the protein architecture of hydrolytic enzymes that degrade polysaccharides, one or more non-catalytic CMBs are present besides the catalytic modules [302,303,304]. The CBMs have been shown to increase the proximity of the enzyme to its substrate, especially for insoluble substrates.

*P. kudriazvevii* is widely distributed in the environment that is used in traditional food and beverage fermentations [305,306]. *P. kudriavzevii* is exceptionally tolerant to stresses and, therefore, has been used to produce bioethanol [307,308], succinic acid [309], and glycerol (under the name *C. glycerinogenes*) [310]. *P. kudriazvevii* can be pathogenic for humans. Recently, an outbreak of fungaemia in a neonatal intensive care unit due to *P. kudriavzevii* (a teleomorph of *C. krusei*) was reported [311]. *P. kudriavzevii* can adhere to intestinal cells although this is strongly strain dependent [312,313] as well as to abiotic surfaces such as polystyrene and stainless-steel surfaces [166]. We found two Flo11-type adhesins in the Pfam database: one that contains only one Flo11 domain and one that contains a Flo11 domain in the N-terminal region of the protein as well as two CMB_1 domains (Table 3).

*Wickerhamomycesanomalus* is a heterothallic, ascomycetous yeast, forming one to four hat-shaped ascospores [314]. *W. anomalus* (synonym *C. pelliculosa*, formerly also known as *Pichia anomala* and *Hansenula anomala*) was recently assigned to the genus *Wickerhamomyces* based on phylogenetic analysis of gene sequences [315]. This species has a wide biotechnological potential [316] for its use in the fermentation of wines [317,318], as a biopreservation or biocontrol agent, and the production of biofuels and therapeutic molecules used in human medicine [319,320,321]. It shows a wide spectrum antimicrobial activity, being active against a variety of microorganisms including other yeasts, filamentous fungi and bacteria [316]. *W. anomalus* is a very rare pathogen causing blood stream infections in neonates [275,322,323,324,325]. It has been shown that *W. anomalus* can form biofilms on abiotic surfaces of a brewery plant [326] as well as on polystyrene and steel surfaces in vitro [166].

*Lodderomyces elongisporus* has been isolated from a variety of fruit concentrates, fresh fruits and soft drinks, wine [327] and insects [328]. It was considered as a teleomorph of *C. parapsilosis*, but 18S rRNA sequencing revealed that *L. elongisporus* represents a distinct species [329,330]. Recently, it was identified in catheter-related bloodstream infections, where it was isolated from both blood and catheter tip cultures from patients [60,331,332,333,334,335,336,337]. The adhesion of *L. elongisporus* to polystyrene and steel surfaces in vitro has been demonstrated [166]. We found one Flo11-type adhesin that contains one Flo11 domain in the N-terminal region of the protein as well as two flocculin type 3 repeats, in the Pfam database (Table 3).

*Hyphopichia burtonii* is a yeast that is responsible for the spoilage of food bakery products, cookies and cured meat [338,339]. It has been reported causing cutaneous infection in Barbastelle bats [340]. Recently, the first human infection caused by *H. burtonii*, resulting in peritonitis in a patient on peritoneal dialysis initially diagnosed as sterile peritonitis, resulting in delayed diagnosis and treatment [341]. We found one Flo11-type adhesin that contains one Flo11 domain in the N-terminal region of the protein as well as one flocculin type 3 repeats, in the Pfam database (Table 3).

*Debaryomyces hansenii* (*Torulaspora hansenii*) (teleomorph *C. famata*) is a hemiascomycetous marine yeast that is usually found in natural substrates and in various cheese due to its high tolerance to salt and growth at low temperatures [342]. It has also been detected in human infections [343,344,345]. However, *C. guilliermondii* and other *Candida* have been misidentified as *D. hansenii/C. famata* [346,347] since they are extremely difficult to differentiate phenotypically [348,349]. The adhesion of *D. hansenii* to intestinal cells has been demonstrated [312]. One Flo11-type adhesin that contains one Flo11 domains in the N-terminal region, was identified in the Pfam database (Table 3).

### 4.2. Non-Pathogenic S. cerevisiae Encountered in Rare Infections

The Flo adhesins were first discovered in *S. cerevisiae* and *S. pastorianus.* They have been studied intensely, mainly due to their role during the beer fermentation process. *S. cerevisiae* and *S. patorianus* (Table 2) can be found in many natural environments including fruits, trees, and soil, and has been used for centuries for the production of beer [63,350], wine, and bread. *S. cerevisiae* is designated as a GRAS (“Generally Recognized as Safe”) yeast. Despite its ubiquity and long association with humans, *S. cerevisiae* is rarely implicated as a causative agent of infections in healthy individuals. *S. cerevisiae* can be present in the skin, oral cavities, oropharynx, duodenal mucosa, digestive tract and vagina of healthy persons [351,352,353]. However, *S. cerevisiae* has been recognized as an emerging fungal pathogen for immunocompromised individuals in recent decades [354]. Since the 1990s [351], there have been a growing number of reports about *S. cerevisiae* invasive infections, and novel strains continue to be developed [355,356]. *S. cerevisiae* infections have been often associated with administration of probiotic *S. cerevisiae* var. *boulardii* strains or certain strains of *S. cerevisiae* [357,358,359,360,361,362,363,364,365,366] (Table 2).

*S. cerevisiae* adhesion is the first step in the infection process. Adhesion can be on biotic or abiotic surfaces such as plastics, stainless steel or glass surfaces, which can lead to the development of biofilms [45,129,130]. The rise in systemic fungal infections does also coincide with the increasing use of implants such as plastic catheters, prosthetic heart valves, cardiac pacemakers, endotracheal tubes, dentures, and cerebrospinal fluid shunts [367,368]. Access of fungi to the bloodstream and internal organs results from their ability to adhere to these prostheses, and next, to various surface receptors of host tissue cells. Prostheses can also serve as a carrier for fungal biofilms and thus provide an internal reservoir of highly resistant infective cells. It has been demonstrated that the adhesion to abiotic surfaces is mediated by Flo11p (Table 1) (see also Section 3.2) [69]. It is well known that microorganisms growing as biofilms are more resistant to various drugs and treatments than solitary cells and biofilms show an enhanced protection from host defences [369,370]. The involvement of the lectin-type Flo proteins in the development of *S. cerevisiae* biofilms has not yet been described. Based on the binding mechanism of the lectin-type Flo proteins, i.e., binding to mannose containing glycans (i.e., mannose, Manα-1,2-Man, Manα-1,3-Man and Manα-1,6-Man glycan determinants), it can be hypothesized that *S. cerevisiae* cells expressing these flocculins could bind to cell receptors with high mannose N-glycans.

*S. boulardii* (Table 2) is a probiotic yeast that is often used for the treatment of gastrointestinal (GI) tract disorders such as diarrhea symptoms or chronic diseases such as inflammatory bowel disease [365,371,372]. Even though *S. boulardii* is generally regarded as safe [373], fungemia and sepsis are possible concerns, particularly in immunocompromised patients [371]. Rare occurrences of fungemia have been reported in people receiving therapeutic doses of *S. boulardii* or post-surgery [357,358,368,374,375]. These cases make it evident that *S. boulardii* fungemia is a distinct but rare possibility in patients with severely compromised health conditions, especially those involving the GI tract or the circulatory system [371]. *S. boulardii* and *S. cerevisiae* are genetically very similar, each containing 16 chromosomes with greater than 99% relatedness by average nucleotide identity [376]. One of the important differences include the genes expressing some flocculation proteins, which contribute to a different adhesion profile of *S. boulardii* when compared to *S. cerevisiae* [377]. Complete flocculin genes were identified in the whole genomes of *S. boulardii* where the repeats and their copies were varying even within *S. boulardii* genomes [376]. *S. boulardii* harbours the flocculin genes *FLO1*, *FLO10* and *FLO11* as well as the (non-truncated) *FLO8* gene involved in the regulation of the expression of the *FLO* genes. These genes are located at telomeres and are highly repetitive, and the maximum number of repeats identified in the strains of *S. boulardii* could be conferring higher adhesive properties to the organism.

## 5. Conclusions

Yeast adhesion proteins play a fundamental role in many processes where cell-cell or cell-substrate interactions are involved such as switching from a unicellular lifestyle to a multicellular one. They are also critical in pathogenic yeast-host interactions. The first step in the infection of fungal pathogens in humans is the adhesion of the pathogen to host tissue cells or abiotic surfaces such as catheters. One of the main players involved in this are the expressed cell wall adhesins. Here, we reviewed the Flo adhesins that could be involved in human yeast infections.

The Flo adhesin family was originally subdivided into two subgroups. Based on the recent knowledge of the protein architecture of the Flo adhesins, we redefined these two subgroups into a PA/GLEYA Flo adhesin class and a Flo11-type class. These both classes are further subdivided according to the presence of 1 or more additional “Flo” domains. In this way, the PA14/GLEYA Flo adhesin class could be further subdivided into adhesins that besides a PA14 domain or GLEYA domain also contains a flocculin domain or/and a flocculin type 3 repeat domain. The Flo11-type adhesins could be further subdivided into architectures containing only the Flo11 domain, the Flo 11 domain and the flocculin domain or the flocculin type 3 repeat, and the Flo 11 domain and another adhesin structural domain.

Pfam database mining based on this new definition of the Flo adhesin family, identified Flo adhesin architectures in many pathogenic yeasts such as several emerging non-*albicans Candida* species that are becoming a major threat to humans due to their resistance to antifungals and high mortality. In some cases, a critical role of the identified adhesin has been elucidated during recent research. However, in many cases the role of these adhesins in the infections process has still to be unravelled. For a detailed understanding of binding mechanism of these adhesins and to develop strategies to inhibit them, the atomic structure of the adhesins should be solved. Until now, the structures of only a few yeast adhesins involved in human infections have been solved (see also [86]). Structural studies will have to be complemented with biophysical interaction studies on the molecular and cellular level. Additionally, the contribution of the Flo adhesins in relation to other expressed adhesins should be investigated to elucidate its role in the overall adhesion process of the infection.

## Figures and Tables

**Table 1:** Protein structures of flocculation adhesins deposited in the Protein Data Bank (PDB, ww.rcsb.org accessed on 2 September 2021).

Flo Adhesin Class/Subtype	Adhesin	Micro-Organism	Ligand in the Structure	Mutations	PDB Code	Interacting Substrate/Function Properties	Refs
Flo-type/PA14-	N-Flo1p	*S. cerevisiae*	Apo	-	4LHL	Cell-cell interaction via cell surface mannans	[93]
			Man	-	4LHN		[93]
Flo-type/PA14	N-Lg-Flo1p	*S. pastorianus*	Apo	-	4GQ7	Cell-cell interaction via cell surface mannans and phospho-mannans	[96]
			Manα-1,2-Man	-	4LHK		[93]
Flo-type/PA14	N-Flo5p	*S. cerevisiae*	Apo	-	2XJQ	Cell-cell interaction via cell surface mannans	[97]
			Man	-	2XJP		[97]
			Man_3_(D1)	-	2XJT		[97]
			Man_5_(D2-3)	-	2XJR		[97]
			Manα-1,2-Man	-	2XJS		[97]
			Manα-1,2-Man	S277A	2XJU		[97]
			Glc	-	2XJV		[97]
Flo-type/GLEYA	N-Epa1p	*C. glabrata*	Gal	-	4A3X	Epithelial cells, fibronectin, mucin	[92]
			Galβ-1,3-Glc	-	4AF9		[95]
			Galβ-1,3-Glc	E227D, Y228N	4AFC		[95]
			Galβ-1,3-GalNAc (T antigen)	-	4ASL		[95]
			Galβ-1,3-GalNAc (T antigen)	-	4D3W		[98]
			Galβ-1,4-Glc (lactose)	-	4COU		[98]
			Glycerol	E227D, Y228N, D229N	4AFA		[95]
			Glycerol	R226I, E227G, Y228K	4AFB		[95]
	N-Epa6p	*C. glabrata*	Galβ-1,4-Glc (lactose)	-	4COU	Epithelial cells	[98]
			Galβ-1,3-GalNAc (T-antigen)	-	4COW		[98]
			N-acetyl-D-lactosamine	-	4COY		[98]
			Lacto-N-biose	-	4COZ		[98]
			α1-3-galactobiose	-	4COV		[98]
	N-Epa9p	*C. glabrata*	Galβ-1,4-Glc	-	4CP0	Epithelial cells	*
			Galβ-1,3-GlcNAc	-	4CP1		*
			Galβ-1,4-GlcNAc	-	4CP2		*
Flo11-type	N-ScFlo11p	*S. cerevisiae*	-	-	4UYR	Cell-cell and cell-hydrophobic plastic adhesion via hydrophobic interactions, biofilm formation, kin discrimination	[69]
			-	-	4UYS		[69]
			-	-	4UYT		[69]
Flo11-type	N-KpFlo11	*K. pastoris ^#^*	-	-	5FV5	Cell-cell adhesion interactions, biofilm formation, kin discrimination	[99]
			Glycerol	-	5FV6		

* Deposited in PDB but not yet published. *^#^ Komagataella pastoris.*

**Table 2:** Examples of fungi expression Flo adhesins of the Flo-type class and adhesin architecture with indication of pathogenic fungi (From Pfam and InterPro database).

Flo Adhesin Subtype	Adhesin Uniprot Entry Pfam Protein Architecture	Description Uniprot	Adhesin Domain [Number of Repeats]	Flocculin [Number of Repeats]	Flocculin_t3 [Number of Repeats]	Organism NCBI Taxonomy ID
PA14	FLO1_YEAST https://www.uniprot.org/uniprot/P32768 (accessed on 24 October 2021)	Flocculation protein Flo1p	1	18	0	*Saccharomyces cerevisiae* S288c 559292 https://www.uniprot.org/taxonomy/559292 (accessed on 24 October 2021)
	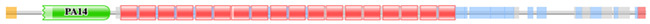					
	FLO10_YEAST https://www.uniprot.org/uniprot/P36170 (accessed on 24 October 2021)	Flocculation protein Flo10p	1	0	2	*Saccharomyces cerevisiae* S288c 559292 https://www.uniprot.org/taxonomy/559292 (accessed on 24 October 2021)
	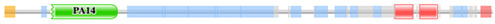					
	FLO5_YEAST https://www.uniprot.org/uniprot/P38894 (accessed on 24 October 2021)	Flocculation protein Flo5p	1	8	3	*Saccharomyces cerevisiae* S288c 559292 https://www.uniprot.org/taxonomy/559292 (accessed on 24 October 2021)
	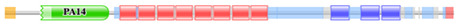					
	FLO9_YEAST https://www.uniprot.org/uniprot/P39712 (accessed on 24 October 2021)	Flocculation protein Flo9p	1	13	3	*Saccharomyces cerevisiae* S288c 559292 https://www.uniprot.org/taxonomy/559292 (accessed on 24 October 2021)
	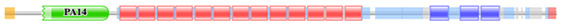					
	B3IUA8_SACPS https://www.uniprot.org/uniprot/B3IUA8 (accessed on 24 October 2021)	Flocculation protein Lg-Flo1p	1	8	3	*Saccharomyces pastorianus* 27292 https://www.uniprot.org/taxonomy/27292 (accessed on 24 October 2021)
	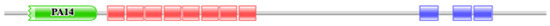					
	A0A0L8VPM7_9SACH https://www.uniprot.org/uniprot/A0A0L8VPM7 (accessed on 24 October 2021)	YHR213Wp flocculin-like protein	1	1	0	*S. cerevisiae* var. *boulardii*
	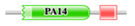					
GLEYA	Q6FR82_CANGA https://www.uniprot.org/uniprot/Q6FR82 (accessed on 24 October 2021)	PA14 domain-containing protein, Epa23p	1	5	0	*Candida glabrata* 284593 https://www.uniprot.org/taxonomy/284593 (accessed on 24 October 2021)
	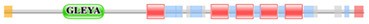					
	C4Y9G0_CLAL4 https://www.uniprot.org/uniprot/C4Y9G0 (accessed on 24 October 2021)	PA14 domain-containing protein	1	0	1	*Clavispora lusitaniae* 306902 https://www.uniprot.org/taxonomy/306902 (accessed on 24 October 2021)
	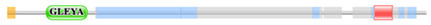					
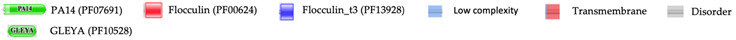						

**Table 3:** Examples of fungi expression Flo adhesins of the Flo11-type class and adhesin architecture with indication of pathogenic fungi (Pfam database, InterPro database).

Adhesin Uniprot Entry Pfam Protein Architecture	Description Uniprot	Flo11 Domain [Number of Repeats]	Flocculin [Number of Repeats]	Flocculin_t3 [Number of Repeats]	Other Adhesin Domain [Number] [Pfam ID]	Organism NCBI Taxonomy ID
A0A1L0DFL1_9ASCOhttps://www.uniprot.org/uniprot/A0A1L0DFL1 (accessed on 24 October 2021)	CIC11C00000002180	1	0	0	—	*Candida intermedia* 45354https://www.uniprot.org/taxonomy/45354 (accessed on 24 October 2021)
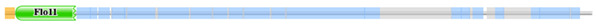						
A0A367Y9C2_9ASCOhttps://www.uniprot.org/uniprot/A0A367Y9C2 (accessed on 24 October 2021)	Cell wall protein RTB1	1	0	0	—	*Candida viswanathii* 5486https://www.uniprot.org/taxonomy/5486 (accessed on 24 October 2021)
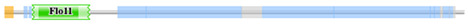						
C4YAK2_CLAL4https://www.uniprot.org/uniprot/C4YAK2 (accessed on 24 October 2021)	Flo11 domain-containing protein	1	0	0	—	*Clavispora lusitaniae* 306902https://www.uniprot.org/taxonomy/306902 (accessed on 24 October 2021)
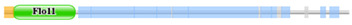						
A0A1V2L8G6_CYBFAhttps://www.uniprot.org/uniprot/A0A1V2L8G6 (accessed on 24 October 2021)	Flocculation protein Flo11p	2	0	0	—	*Cyberlindnera fabianii* 36022https://www.uniprot.org/taxonomy/36022 (accessed on 24 October 2021)
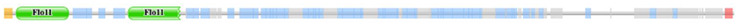						
Q6BXK5_DEBHAhttps://www.uniprot.org/uniprot/Q6BXK5 (accessed on 24 October 2021)	DEHA2B02222p	1	0	0	—	*Debaryomyces hansenii* 284592https://www.uniprot.org/taxonomy/284592 (accessed on 24 October 2021)
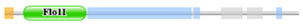						
A5DNK5_PICGUhttps://www.uniprot.org/uniprot/A5DNK5 (accessed on 24 October 2021)	Flo11 domain-containing protein	1	0	0	—	*Meyerozyma guilliermondii* 294746https://www.uniprot.org/taxonomy/294746 (accessed on 24 October 2021)
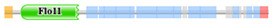						
A0A2U9R2I2_PICKUhttps://www.uniprot.org/uniprot/A0A2U9R2I2 (accessed on 24 October 2021)	Flo11 domain-containing protein	1	0	0	—	*Pichia kudriavzevii* 4909https://www.uniprot.org/taxonomy/4909 (accessed on 24 October 2021)
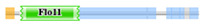						
FLO11_YEASThttps://www.uniprot.org/uniprot/P08640 (accessed on 24 October 2021)	Flocculation protein Flo11p	1	0	0	—	*Saccharomyces cerevisiae* 559292https://www.uniprot.org/taxonomy/559292 (accessed on 24 October 2021)
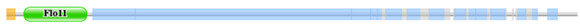						
A0A1L0BS11_9ASCOhttps://www.uniprot.org/uniprot/A0A1L0BS11 (accessed on 24 October 2021)	CIC11C00000003144	6	0	5	—	*Candida intermedia* 45354https://www.uniprot.org/taxonomy/45354 (accessed on 24 October 2021)
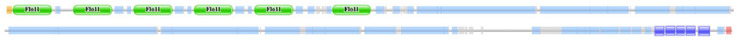						
G8B9T8_CANPChttps://www.uniprot.org/uniprot/G8B9T8 (accessed on 24 October 2021)	Flo11 domain-containing protein	1	0	2	—	*Candida parapsilosis* 578454https://www.uniprot.org/taxonomy/578454 (accessed on 24 October 2021)
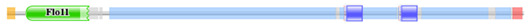						
C5M338_CANTThttps://www.uniprot.org/uniprot/C5M338 (accessed on 24 October 2021)	Flo11 domain-containing protein	1	0	2	—	*Candida tropicalis* 294747https://www.uniprot.org/taxonomy/294747 (accessed on 24 October 2021)
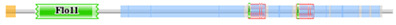						
C4XZ24_CLAL4https://www.uniprot.org/uniprot/C4XZ24 (accessed on 24 October 2021)	Uncharacterized protein	3	0	4	*—*	*Clavispora lusitaniae* 306902https://www.uniprot.org/taxonomy/306902 (accessed on 24 October 2021)
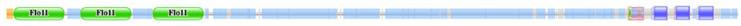						
A0A1E4RJE3_9ASCOhttps://www.uniprot.org/uniprot/A0A1E4RJE3 (accessed on 24 October 2021)	Flo11 domain-containing protein	1	0	1	—	*Hyphopichia burtonii* 984485https://www.uniprot.org/taxonomy/984485 (accessed on 24 October 2021)
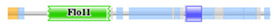						
A5E4F6_LODELhttps://www.uniprot.org/uniprot/A5E4F6 (accessed on 24 October 2021)	Flo11 domain-containing protein	1	0	2	—	*Lodderomyces elongisporus* 379508https://www.uniprot.org/taxonomy/379508(accessed on 24 October 2021)
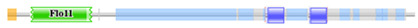						
A5DGW9_PICGUhttps://www.uniprot.org/uniprot/A5DGW9 (accessed on 24 October 2021)	Flo11 domain-containing protein	1	0	1	*—*	*Meyerozyma guilliermondii* 294746https://www.uniprot.org/taxonomy/294746 (accessed on 24 October 2021)
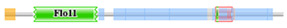						
A0A2H1A319_CANARhttps://www.uniprot.org/uniprot/A0A2H1A319 (accessed on 24 October 2021)	Flo11 domain-containing protein	1	0	0	Collagen ^1^ 1 PF01391	*Candida auris* 498019https://www.uniprot.org/taxonomy/498019 (accessed on 24 October 2021)
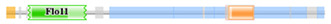						
A0A2V1B0R1_9ASCOhttps://www.uniprot.org/uniprot/A0A2V1B0R1 (accessed on 24 October 2021)	Flo11 domain-containing protein	1	0	0	Collagen ^1^ 1 PF01391	*Candida haemuloni* 45357https://www.uniprot.org/taxonomy/45357 (accessed on 24 October 2021)
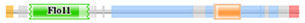						
A0A4T0 × 4H6_9ASCOhttps://www.uniprot.org/uniprot/A0A4T0X4H6 (accessed on 24 October 2021)	Uncharacterized protein	1	0	0	CMB_1 ^2^ 3 PF00734	*Candida inconspicua* 52247https://www.uniprot.org/taxonomy/52247 (accessed on 24 October 2021)
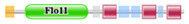						
A5E3T4_LODELhttps://www.uniprot.org/uniprot/A5E3T4 (accessed on 24 October 2021)	Flo11 domain-containing protein	1	0	0	Candida_ALS ^3^ 20 PF05792	*Lodderomyces elongisporus* 379508https://www.uniprot.org/taxonomy/379508 (accessed on 24 October 2021)
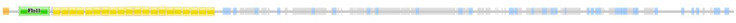						
A0A099NZM2_PICKUhttps://www.uniprot.org/uniprot/A0A099NZM2 (accessed on 24 October 2021)	Flocculation protein Flo11p	1	0	0	CMB_1 ^2^ 2 PF00734	*Pichia kudriavzevii* 4909https://www.uniprot.org/taxonomy/4909 (accessed on 24 October 2021)
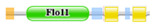						
A0A1E3NVU9_WICAAhttps://www.uniprot.org/uniprot/A0A1E3NVU9 (accessed on 24 October 2021)	Uncharacterized protein	1	0	0	CMB_1 ^2^ 2 PF00734	*Wickerhamomyces anomalus* 683960https://www.uniprot.org/taxonomy/683960 (accessed on 24 October 2021)
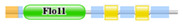						
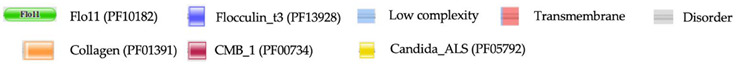						

^1^ Collagen: Collagen triple helix repeat; ^2^ CMB_1: Carbohydrate-binding module, fungal cellulose binding domain; ^3^ Candida_ALS: Candida agglutinin-like (ALS).

## Data Availability

Not applicable.
